# Chitooligosaccharide and Its Derivatives: Preparation and Biological Applications

**DOI:** 10.1155/2014/654913

**Published:** 2014-03-03

**Authors:** Gaurav Lodhi, Yon-Suk Kim, Jin-Woo Hwang, Se-Kwon Kim, You-Jin Jeon, Jae-Young Je, Chang-Bum Ahn, Sang-Ho Moon, Byong-Tae Jeon, Pyo-Jam Park

**Affiliations:** ^1^Department of Biotechnology, Konkuk University, Chungju 380-701, Republic of Korea; ^2^Department of Applied Life Science, Konkuk University, Chungju 380-701, Republic of Korea; ^3^Specialized Graduate School of Convergence Science and Technology, Department of Marine Bioconvergence Science, Busan 608-737, Republic of Korea; ^4^School of Marine Biomedical Sciences, Jeju National University, Jeju 690-756, Republic of Korea; ^5^Department of Marine Bio-Food Sciences, Chonnam National University, Yeosu 550-749, Republic of Korea; ^6^Division of Food and Nutrition, Chonnam National University, Gwangju 550-757, Republic of Korea; ^7^Nokyong Research Center, Konkuk University, Chungju 380-701, Republic of Korea

## Abstract

Chitin is a natural polysaccharide of major importance. This biopolymer is synthesized by an enormous number of living organisms; considering the amount of chitin produced annually in the world, it is the most abundant polymer after cellulose. The most important derivative of chitin is chitosan, obtained by partial deacetylation of chitin under alkaline conditions or by enzymatic hydrolysis. Chitin and chitosan are known to have important functional activities but poor solubility makes them difficult to use in food and biomedicinal applications. Chitooligosaccharides (COS) are the degraded products of chitosan or chitin prepared by enzymatic or chemical hydrolysis of chitosan. The greater solubility and low viscosity of COS have attracted the interest of many researchers to utilize COS and their derivatives for various biomedical applications. In light of the recent interest in the biomedical applications of chitin, chitosan, and their derivatives, this review focuses on the preparation and biological activities of chitin, chitosan, COS, and their derivatives.

## 1. Introduction

Synthetic polymers are gradually being replaced by biodegradable materials especially those derived from replenishable, natural resources [1]. Natural biopolymers have several advantages, such as availability from replenishable agricultural or marine food resources, biocompatibility, and biodegradability, thereby leading to ecological safety and the possibility of preparing a variety of chemically or enzymatically modified derivatives for specific end uses. Polysaccharides, as a class of natural macromolecules, have the tendency to be extremely bioactive and are generally derived from agricultural feedstock or crustacean shell wastes [2].

Chitosan is a natural nontoxic biopolymer produced by the deacetylation of chitin, a major component of the shells of crustaceans such as crab, shrimp, and crawfish; chitooligosaccharides (COS) are the degraded products of chitosan or chitin prepared by enzymatic or chemical hydrolysis of chitosan.

Chitosan and its derivatives have shown various functional properties that have made them possible to be used in many fields including food [3], cosmetics [4], biomedicine [5], agriculture [6], environmental protection [7], and wastewater management [8]. Furthermore, biodegradable, nontoxic, and nonallergenic nature of chitosan especially encourages its potential use as a bioactive material [9]. Even though chitosan is known to have important functional activities, poor solubility makes them difficult to use in food and biomedicinal applications. Unlike chitosan, its hydrolyzed products and COS are readily soluble in water due to their shorter chain lengths and free amino groups in D-glucosamine units [10]. The low viscosity and greater solubility of COS at neutral pH have attracted the interest of many researchers to utilize chitosan in its oligosaccharide form. Especially, research on COS in food and nutrition fields has emphasized their ability to improve food quality and human health progression.

In light of the recent interest in the biomedical applications of chitin, chitosan, and its derivatives, this review focuses on the preparation and biological activities of chitin, chitosan, COS, and their derivatives.

## 2. Chitin

**Figure 1:**
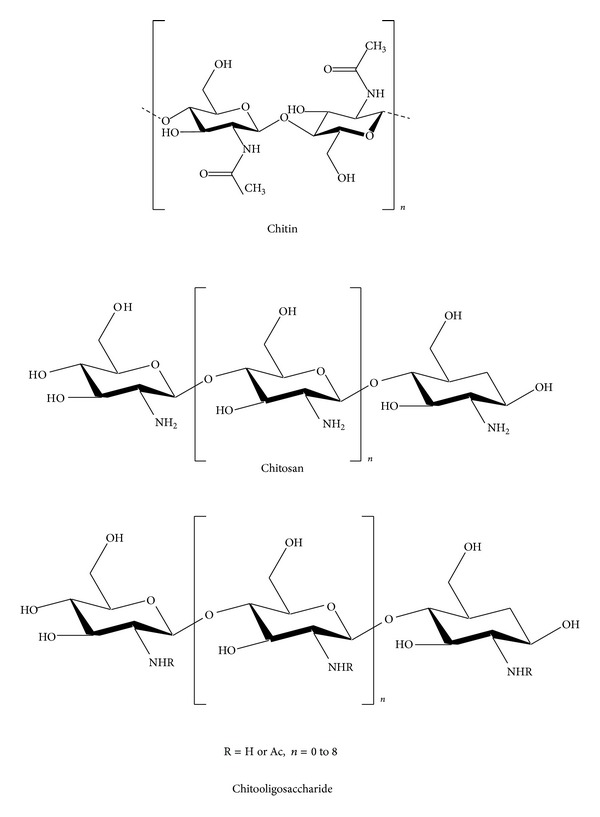


Chitin (Figure 1), a mucopolysaccharide and the supporting material of crustaceans and insects, is the second most abundant polymer after cellulose found in nature; it is produced by many living organisms and is present usually in a complex with other polysaccharides and proteins. Chitin was found as a major component in arthropods (insects, crustaceans, arachnids, and myriapods), nematodes, algae, and fungi [11–14].

Its immunogenicity is exceptionally low in spite of the presence of nitrogen. It is highly insoluble material resembling cellulose with its solubility and low chemical reactivity. It may be regarded as cellulose with hydroxyl at position C-2 replaced by an acetamido group. Like cellulose, it functions naturally as a structural polysaccharide. It is white, hard inelastic nitrogenous polysaccharide [15, 16].

Chitin is a linear polysaccharide composed of (1 → 4) linked 2-acetamido-2-deoxy-*β*-d-glucopyranosyl units and occurs naturally in three polymorphic forms with different orientations of the microfibrils, known as *α*-, *β*-, and *γ*-chitin [1, 17]. The *α*-form has antiparallel chains and is a common and the most stable polymorphic form of chitin in nature, which is prevalent in crustaceans and in insect chitinous cuticles [18–20]. The *β*-form of chitin is rare; it occurs in pens of mollusks and is characterized by a loose-packing parallel chains fashion with weak intermolecular interactions and higher solubility and swelling than *α*-form; *β*-chitin was prepared from the pens of the squid *Ommastrephes bartrami* [21, 22], *Loligo* species, and cuttlefish (*Sepia officinalis*) [18, 23–25]. The *γ*-form is characterized by a mixture of antiparallel and parallel chains and was found in the cocoons of insects [26].

Besides its application as a starting material for the synthesis of chitosan and chitooligosaccharides, chitin itself has been a center of many therapeutic applications and is thought to be a promising biomaterial for tissue engineering and stem cell technologies [27].

Bae et al. [28] demonstrated that oral administration of chitin (*α* and *β* forms) is beneficial in preventing food allergies; the oral administration of chitin was accomplished by milling it to particle size less than 20 *μ*m and mixing it with feed. Their results showed that *α*-form reduced serum levels of peanut-specific IgE and both the forms decreased the levels of interleukins (IL), IL-5 and IL-10, and increased the levels of IL-12. Dietary supplementation of chitin has shown to exert positive immunomodulatory effects [29, 30]; antibacterial activity of chitin, prepared from shrimp shell waste, was reported by Benhabiles et al. [31].

## 3. Chitosan

Chitosan [poly-(*β*-1/4)-2-amino-2-deoxy-D-glucopyranose] is a natural nontoxic biopolymer produced by the deacetylation of chitin. Chitosan (Figure 1) has three types of reactive functional groups, an amino group as well as both primary and secondary hydroxyl groups at the C-2, C-3, and C-6 positions, respectively. Chemical modifications of these groups have provided numerous useful materials in different fields of application. Currently, chitosan has received considerable attention for its commercial applications in the biomedical, food, and chemical industries [9, 32–36].

Chitosan solubility, biodegradability, reactivity, and adsorption of many substrates depend on the amount of protonated amino groups in the polymeric chain and thereby on the proportion of acetylated and nonacetylated glucosamine units. The amino groups (pKa from 6.2 to 7.0) are completely protonated in acids with pKa smaller than 6.2 making chitosan soluble. Chitosan is insoluble in water, organic solvents, and aqueous bases and it is soluble after stirring in acids such as acetic, nitric, hydrochloric, perchloric, and phosphoric [37–39].

Applications of chitin are limited compared to chitosan because chitin is chemically inert and is insoluble in both water and acid, while chitosan is relatively reactive and can be produced in various forms. Chitosan is normally insoluble in neutral or basic pH conditions while being soluble in acidic pH. The solubility of chitosan depends upon the distribution of free amino and N-acetyl groups. In dilute acids (pH < 6), the free amino groups are protonated and the molecule becomes soluble [40].

Due to its unique biological characteristics, including biodegradability and nontoxicity, many applications have been found either alone or blended with other natural polymers (starch, gelatin, and alginates) in the food, pharmaceutical, textile, agriculture, water treatment, and cosmetics industries [41–46].

Chitosan lacks irritant or allergic effects and is biocompatible with both healthy and infected human skin [47]. When chitosan was administered orally in mice, the LD_50_ was found to be in excess of 16 g/kg, which is higher than that of sucrose [48]. The intriguing properties of chitosan have been known previously and the polymer has been used in the fields of agriculture, industry, and medicine. In agriculture, chitosan has been described as a plant antivirus, an additive in liquid multicomponent fertilizers [49], and it has also been investigated as a metal-recovering agent in agriculture and industry [50]. Chitosan has been noted for its application as a film-forming agent in cosmetics [51], a dye binder for textiles, a strengthening additive in paper [52], and a hypolipidic material in diets [53]. It has been used extensively as a biomaterial [54], owing to its immunostimulatory activities [55], anticoagulant properties [56], antibacterial and antifungal action [57], and its action as a promoter of wound healing in the field of surgery [58].

Chitosan and its derivatives possess some special properties for use in regenerative medicine. Several studies have examined the host tissue response to chitosan based implants. In general, these materials are nontoxic and biodegradable with living tissues and evoke a minimal foreign body reaction with little or no fibrous encapsulation [59].

Antimicrobial activity of chitosan has been demonstrated against many bacteria, filamentous fungi, and yeasts [60–63]. Chitosan has wide spectrum of activity and high killing rate against Gram-positive and Gram-negative bacteria but lower toxicity toward mammalian cells [64, 65].

Owing to the presence of hydroxyl, amine, and acetylated amine groups, chitosan, low molecular weight chitosan, and COS interact readily with various cell receptors that trigger a cascade of interconnected reactions in living organisms resulting in anti-inflammatory [66], anticancerogenic [67], antidiabetic [68], antimicrobial [69], anti-HIV-1 [70], antioxidant [71], antiangiogenic [72], neuroprotective [73], and immunostimulative [74] effects.

## 4. Chitooligosaccharides

Chitosans with degrees of polymerization (DPs) <20 and an average molecular weight less than 3900 Da are called chitosan oligomers, chitooligomers, or chitooligosaccharides [75]. COS (Figure 1) are generated by depolymerization of chitin or chitosan using acid hydrolysis, hydrolysis by physical methods and enzymatic degradation [76].

Hydrolysis of chitosan can progress by use of acid as hydrochloric acid, acid with electrolytes, nitrous acid, phosphoric acid, hydrofluoric acid, or oxidative reductive methods with hydrogen peroxide or persulfate. Other acids as lactic acid [77], trichloroacetic acid [78], formic acid [79], and acetic acid have also been studied for their degradative effect on chitin or chitosan. However, due to the complexity of controlling the progress of the reaction, these treatments also result in the formation of secondary compounds that are difficult to remove [75]. With hydrolysis by physical methods such as irradiation with low-frequency ultrasound (20 kHz), partial depolymerization is obtained, reducing the average MW from 2000 kDa down to 450 kDa or from 300 kDa to 50 kDa, but the reduction of molecular weight is limited [75].

Enzymatic methods for the hydrolysis of chitosan involve the use chitosanases and other nonspecific enzymes; this method is performed in gentle conditions and the MW distribution of the product can be controlled [80, 81]. Pantaleone et al. [82] reported the hydrolytic susceptibility of chitosan to a wide range of enzymes, including glycanases, proteases, and lipases derived from bacterial, fungal, mammalian, and plant sources.

Recently, COS have been the subject of increased attention in terms of their pharmaceutical and medicinal applications, due to their nontoxic and high solubility properties as well as their positive physiological effects.

Recent advances have insight into the health benefits of COS, possessing several beneficial biological effects including lowering blood cholesterol, lowering high blood pressure, protective effects against infections, controlling arthritis, improvement of calcium uptake, and enhancing antitumor properties.

The radical scavenging effects of COS were reported by Mendis et al. [83]. They reported that COS successively participated in scavenging intracellular radicals and also suppressed NF-*κ*B activation.

The antitumor activity of COS was first reported in early 1970s [84]. This activity was suggested mainly due to its cationic property exerted by amino groups, and later it was accepted that the molecular weight also plays a major role for the antitumor activity [85]. Some researchers found that antitumor effects of COS were due to increased activity of natural killer lymphocytes as observed in sarcoma 180-bearing mice [86]. There have also been studies showing the apoptotic effect of COS on human hepatocellular carcinoma cells by the upregulation of Bax (Bcl-2-associated X protein) [87]. Pangestuti et al. [88] suggested that COS with molecular weight <1 kDa exhibit potent anti-inflammatory activity and also that treatment with COS results in attenuation of proinflammatory mediators in LPS (lipopolysaccharides) stimulated BV2 microglia by MAPK (mitogen activated protein kinases) signaling pathway. Administration of COS has been found to be effective against cyclophosphamide induced immunosuppression in mice [89]. The study of Mei et al. [89] also suggested that COS may also be effective in enhancing systemic immune responses and in modulating the functions of immunocompetent cells.

Wei et al. [90] showed that a high concentration of COS significantly proliferated the mice marrow cells and induced CD34+ cells into megakaryocyte progenitor cells. However, in their study, COS could not enhance the proliferation of CD19+ and CD4+ and promote CD34+ cells to differentiate into lymphoid progenitor cells suggesting that COS can promote hematopoietic stem/progenitor cells hyperplasia in mice.

Senevirathne et al. [91] studies on the effect of COS on tert-butyl hydroperoxide (t-BHP) induced damage in Chang liver cells showed that COS protected Chang liver cells against oxidative damage induced by t-BHP via inhibiting production of ROS and lipid peroxidation and the elevation of the levels of antioxidant enzymes.

The inhibitory effects of COS on degranulation and cytokine generation in rat basophilic leukemia RBL-2H3 cells were investigated by Vo et al. [92]. Their results indicated that COS contribute to attenuation of allergic reactions. Their study suggested that COS (MW: 1 to 3 kDa) possess the highest inhibitory effects on degranulation and cytokine generation of mast cells. They also suggested that COS with different molecular weight ranges might lead to variable absorption, which corresponds to the different inhibitory effects in mast cells.

Efficacy of COS in the management of diabetes in alloxan induced mice was evaluated by Katiyar et al. [93]. Their study suggested that COS have significant effect of antidiabetic activity, hypolipidemic activity, and antioxidative properties in alloxan induced diabetic mice.

## 5. Chitooligosaccharide Derivatives

### 5.1. Amino Derived COS

COS, hydrolytic products of chitosan, have amino groups or acetamide groups at C-2 position depending on their degree of acetylation. Partly based on these functional groups, they have exhibited a number of biological activities.

**Figure 2:**
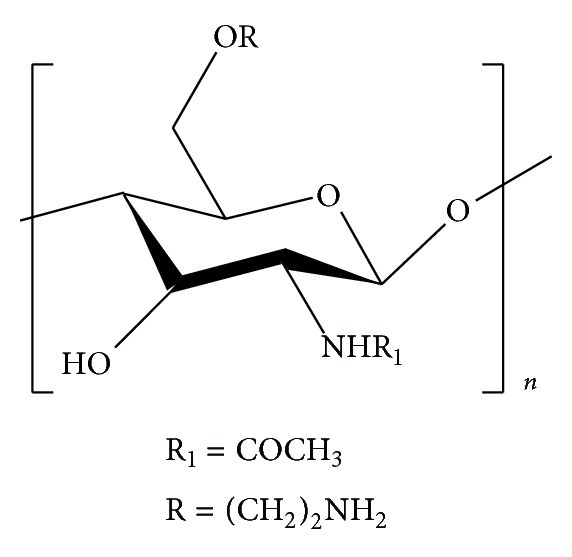
Aminoethyl chitooligosaccharide.

Ngo et al. [94] synthesized aminoethyl COS (AE-COS, Figure 2) by grafting aminoethyl functionality to improve its angiotensin I converting enzyme inhibitor potential. ACE plays an important physiological role in regulating blood pressure by converting an inactive form of decapeptide, angiotensin I, to a potent vasoconstrictor, octapeptide, angiotensin II, and by inactivating catalytic function of bradykinin, which has depressor action. Therefore, inhibition of ACE is considered to be an important therapeutic approach for controlling hypertension [95].

Ngo et al. [94] replaced the hydroxyl group at C-6 position by aminoethyl group because the C-6 hydroxyl groups had the highest reactivity for aminoethylation, while the structure of COS was maintained; the AE-COS thus formed was completely soluble in water. In their study, AE-COS with molecular mass ranging between 800.79 and 4765 Da was prepared from COS with molecular weight 800–3000 Da by grafting aminoethyl groups at C-6 position of pyranose ring and its effects on ACE inhibition were investigated. Their study showed that AE-COS exhibited an 89.3% ACE inhibition at 2.5 mg/mL and its IC50 value was 0.8017 mg/mL.

**Figure 3:**
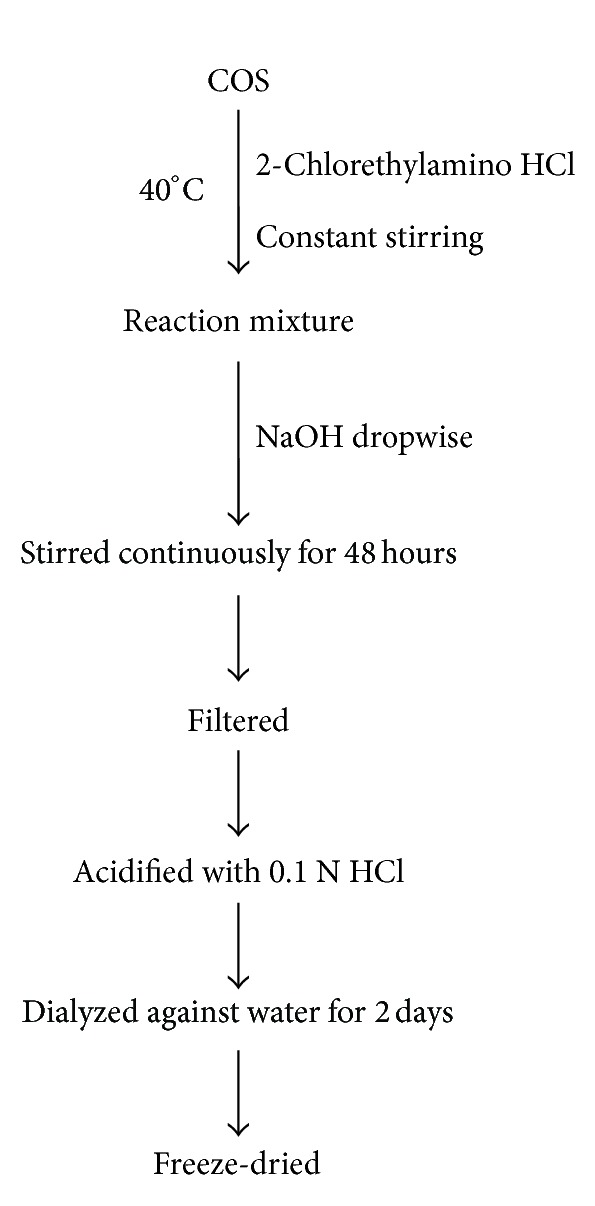
Synthesis of AE-COS.

Ngo et al. [96] investigated the inhibitory effects of AE-COS on oxidative stress in mouse macrophages (RAW 264.7 cells). They prepared AE-COS by adding 2-chlorethylamino hydrochloride to COS while stirring at 40°C. NaOH was added to the reaction mixture dropwise and continuously stirred for 48 h. The solution was then filtered and the reaction mixture was acidified with 0.1 N HCl and dialyzed against water for 2 days. The product was freeze-dried to give AE-COS (Figure 3). The results of Ngo et al. [96] indicated that in HL-60 cells, AE-COS exhibited a greater inhibitory effect (a 20% increase) on myeloperoxidase activity as compared to COS at 100 *μ*g/mL. They also reported that AE-COS can prevent oxidative stress to membrane protein of live cells and exhibits inhibitory effect against DNA oxidation in live cells. Ngo et al. [96] investigated the direct scavenging effects of AE-COS and COS in live cell system. For that, RAW 264.7 cells were labeled with fluorescence probe DCFH-DA (dichlorodihydrofluorescein diacetate), which is the specific probe for reactive oxygen species (ROS) that was used. Their study confirmed that AE-COS has antioxidant activity in dose- and time-dependent manner and has potent direct free radicals scavenging ability in a cellular environment.

The antioxidant and anti-inflammatory activities of AE-COS in murine microglial cells (BV-2) were investigated by Ngo et al. [97]. Neuroinflammation or inflammation of the brain is closely involved in pathogenesis of several neurodegenerative diseases, such as Parkinson's disease, Alzheimer's disease, human immunodeficiency virus-associated dementia, and multiple sclerosis. It was found that the treatment of AE-COS in BV-2 cells at 100 *μ*g/mL inhibited ROS, DNA, protein, and lipid oxidation. AE-COS was also studied for its inhibitory activity against lipopolysaccharide induced inflammatory responses in BV-2 cells. It was found that AE-COS reduced the level of nitric oxide (NO) and prostaglandin E2 production by diminishing the expression of inducible NO synthase and cyclooxygenase-2 without significant cytotoxicity. They also observed that in LPS treated BV-2 cells, AE-COS lowered the levels of TNF-*α* and IL-1*β* in a dose-dependent manner.

Karagozlu et al. [98] synthesized water-soluble amino derivatized COS derivatives, aminoethyl COS (AE-COS), dimethyl aminoethyl COS (DMAE-COS), and diethyl aminoethyl COS (DEAE-COS) and evaluated their apoptotic activity on human stomach adenocarcinoma (AGS cells). Their study showed that exposure of AGS cells to the aminoethylated COS derivatives resulted in the inhibition of cell proliferation in a dose-dependent manner; at 50 and 500 *μ*g/mL, AE-COS inhibited the cell proliferation by 22% and 84%, DMAE-COS by 45% and 85%, and DEAE-COS by 68% and 86%. Karagozlu et al. [98] also suggested that COS and amino derivatized COS derivatives induced apoptosis in AGS cancer cells by mitochondrial pathways, via the upregulation of Bax expression and activation caspases.

Antiproliferative effects of amino derivatized COS on AGS human gastric cancer cells were investigated by Karagozlu et al. [99]. They were able to deduce that exposure of AGS cells to increasing concentrations of amino derivatized COS resulted in a dose- and time-dependent decrease in cell viability relative to control cells and that amino derivatized COS induce cell death in AGS cells through a typical apoptotic pathway. Karagozlu et al. [99] reported that AE-COS induced apoptosis via induction of p53 and p21 and inhibition of Bcl-2 and Bax and DEAE-COS induced apoptosis via induction of p53 and p21, activation of Bax, and inhibition of Bcl-2.

### 5.2. Carboxylated COS

**Figure 4:**
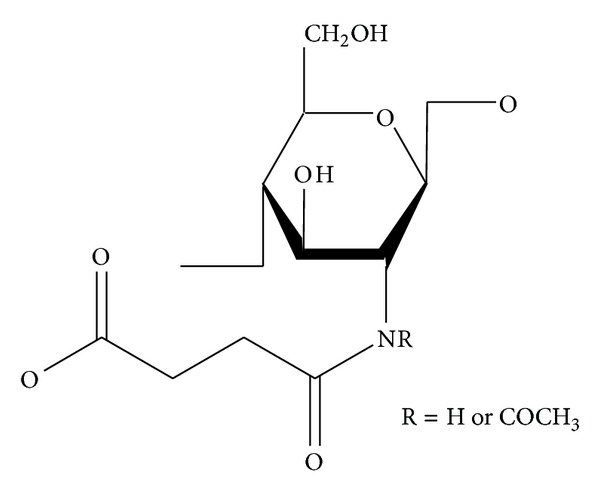
Carboxylated chitooligosaccharide.

**Figure 5:**
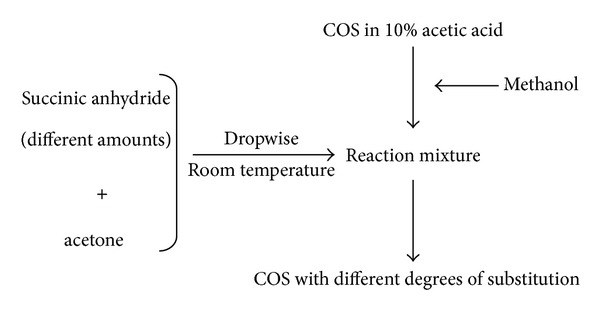
Synthesis of CCOS.

The functional properties of COS derivatives are mainly dependent upon their functional groups and molecular weight. Low molecular weight COS derivatives not only are easily solubilized in aqueous media, but also are preferred to be used for numerous applications. Rajapakse et al. [100] synthesized carboxylated chitooligosaccharides (CCOS; Figure 4) by introducing carboxyl group (COCH_2_CH_2_COO−) to the amino position of pyranose unit. They accomplished this by adding methanol to a solution of COS in 10% acetic acid followed by addition of different amounts of succinic anhydride dissolved in acetone dropwise at room temperature for 1 h, to obtain CCOS with different substitution degrees (Figure 5). Rajapakse et al. [100] evaluated the effect of these synthesized CCOS on MMP-9 (matrix metalloproteinases-9) expression in human fibrosarcoma cells.

MMPs are a structurally related class of zinc-binding proteases (metzincin) which selectively cleave polypeptide bonds in extracellular matrix (ECM) and remodel structural proteins. However, in several disease states pathological tissue degradation and remodeling take place due to over expression and imbalanced activation of MMPs. In cancer, MMP-9 (gelatinase B, 92 kDa) is thought to play a major role in tumor growth, angiogenesis, and metastasis [101]. Rajapakse et al. [100] reported in their study that CCOS exerted a dose- dependent inhibitory effect on MMP-9 in human fibrosarcoma cell line (HT1080); they observed that reduction in MMP-9 expression was due to downregulation of MMP-9 transcription that was mediated via inhibition of AP-1 (activator protein-1) and this inhibition of MMP-9 expression led to inhibition of tumor invasiveness.

Effect of CCOS on ACE inhibition was assessed by Huang et al. [102]. They observed that attachment of carboxyl group to COS was beneficial for ACE inhibition because it enhanced the binding ability of COS to the obligatory active site of the enzyme.

Rajapakse et al. [103] tested the cellular antioxidant effects of CCOS, by assessing the oxidation inhibition potential on cellular biomolecules such as lipids, proteins, and direct scavenging of reactive oxygen species (ROS). Their results indicated that CCOS inhibited membrane lipid peroxidation in a dose-dependent manner and at low concentrations; it also showed a significantly higher inhibition of cellular lipid peroxidation as compared to COS. The study of Rajapakse et al. [103] also demonstrated that CCOS inhibited membrane protein oxidation and myeloperoxidase and their free radical scavenging studies using 2′, 7′-dichlorofluorescein diacetate (DCFH-DA) on RAW264.7 cells showed that oxidation protection effects exerted by CCOS are due to direct scavenging of cellular radicals.

### 5.3. Gallyl COS

Phenolic compounds are some of the most widespread molecules among plant secondary metabolites [104]. These phenolics are rich in some foods and medicinal plants and considered as natural antioxidants [105]. Among them, gallic acid is particularly abundant in processed beverages such as red wine and green tea [106]. It has been reported that gallic acid possesses a wide range of biological activities, including antioxidant, anti-inflammatory, antimicrobial, and anticancer activities [107].

**Figure 6:**
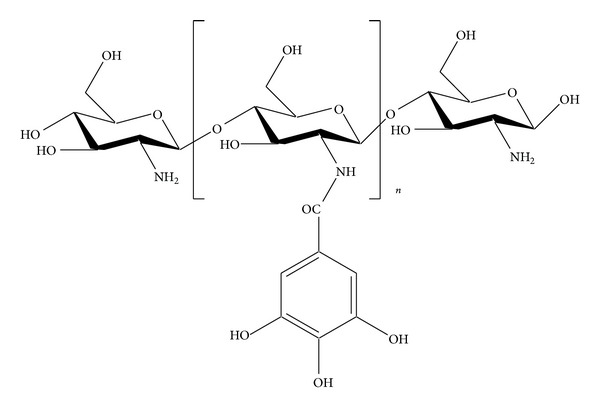
Gallyl chitooligosaccharide.

**Figure 7:**
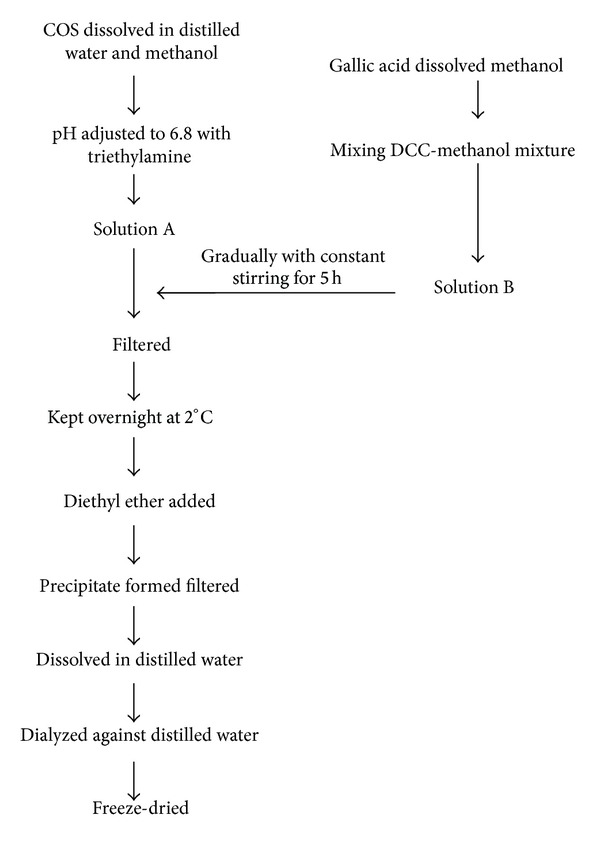
Synthesis of Gallyl COS.

Ngo et al. [108] synthesized gallic acid conjugated COS (G-COS; Figure 6) to evaluate its inhibitory effects on intracellular free radical generation. G-COS was synthesized by mixing two solutions, solution A which was prepared by dissolving COS in distilled water (DW) and methanol and the pH of this solution was adjusted to 6.8 with triethylamine and solution B which was prepared by dissolving gallic acid in methanol and mixing it with DCC- (dicyclohexylcarbodiimide-) methanol mixture. Solution B was gradually added to solution A with constant stirring for 5 hours; the mixture was filtered and kept overnight at 2°C and diethyl ether was added; the precipitate formed was filtered, dissolved in DW, and dialyzed (dialysis membranes molecular weight cutoff below 1 kDa) against DW; the solution was then freeze- dried to obtain G-COS (Figure 7).

Ngo et al. [108] findings suggest that G-COS is a potent scavenger of free radicals and is able to inhibit and prevent oxidative damage to DNA, proteins, and lipids in SW1353 cells; it increased the levels of intracellular antioxidant enzymes (superoxide dismutase (SOD) and glutathione (GSH)) and suppressed the NF-*κ*B activation and expression in H_2_O_2_ induced SW1353 cells, thereby reducing and preventing the oxidative damage to cellular biomolecules in living cells by both indirect and direct ways.

Ngo et al. [109] evaluated the antioxidant effect of gallate chitooligosaccharides in mouse macrophage RAW264.7 cells. They reported that G-COS was able to scavenge cellular radicals in RAW264.7 cells and was able to inhibit oxidative damage to lipids, proteins, and DNA; they also deduced that G-COS could decrease the activation and expression of NF-*κ*B and increase the level of intracellular antioxidant enzymes (SOD and GSH) in oxidative stress induced RAW264.7 cells.

Allergy is considered as a disorder of the immune system in which an exaggerated response occurs when a person is exposed to normally harmless environmental substances, such as animal dander, house dust mites, foods, pollen, insects, and chemical agents [110, 111]. The effect of G-COS on antigen induced allergic reactions in RBL-2H3 mast cells was studied by Vo et al. [112]; they observed the effect of G-COS on the degranulation of mast cells by measuring the amount of histamine released and found that G-COS inhibited the release of histamine in a dose- dependent manner. They concluded that diminished histamine production on treatment with G-COS may be due to the decreased in expression of HDC (L-histidine decarboxylase). The study of Vo et al. [112] also showed that G-COS suppressed intracellular Ca^2+^ elevation and IL-4 and TNF-*α* in antigen stimulated RBL-2H3 mast cells. The suppression in cytokines IL-4 and TNF-*α* might be due to regulation of the MAPKs and NF-*κ*B activation [113]. The overall conclusion of Vo et al. [112] was that the inhibitory effects of G-COS on antigen induced allergic reactions in RBL-2H3 mast cells were shown due to suppression of Fc*ε*RI expression which might cause the inhibition of intracellular signaling activation and subsequent inhibition of intracellular Ca^2+^ elevation, cytokine generation, and histamine release and production. Fc*ε*RI has been well known to play a central role in the induction and maintenance of allergic reactions [114].

### 5.4. Sulfated COS

Glucose sensing is a very important function of pancreatic beta cells and serves in maintaining an appropriate blood glucose level. Impairment of glucose sensing by beta cells results in an alteration in the glucose concentration dependence of insulin secretion in pancreatic beta cells, which may lead to type 2 diabetes [113, 115]. Ishihara et al. [116] characterized the properties of transport, phosphorylation, and utilization of glucose in MIN6 cells and demonstrated that these characteristics are very similar to those in normal pancreatic islets and beta cells.

**Figure 8:**
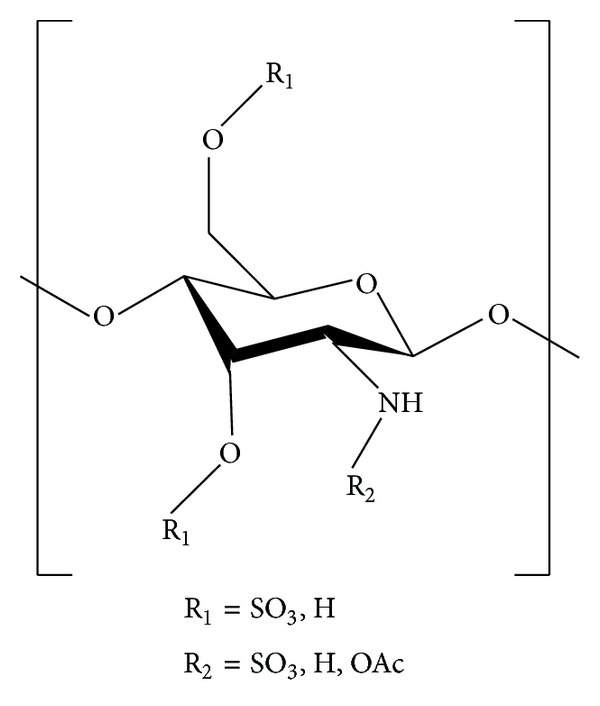
Sulfated chitooligosaccharide.

**Figure 9:**
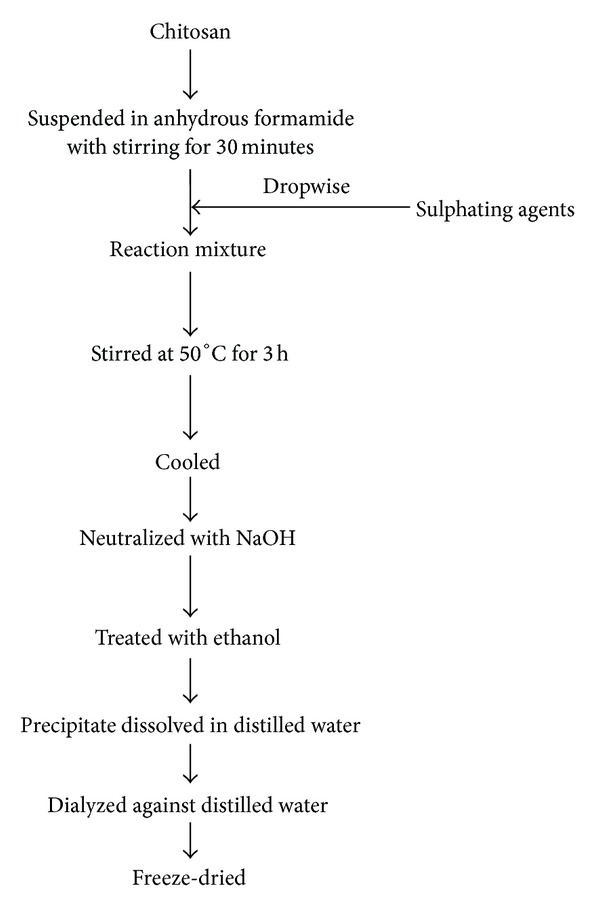
Synthesis of sulfated chitooligosaccharides.

Protective effect of different substituted sulfated COS (COS-S; Figure 8) on MIN6 cells was evaluated by Lu et al. [117]. They synthesized COS-S with different degrees of substitution by using two kinds of sulfating reagent, chlorosulfonic acid and anhydrous formamide in different ratios (1 : 8 and 1 : 12) [117]. Chitosan was suspended in anhydrous formamide by stirring for 30 min, and the sulfating reagents were added drop by drop. The mixture was maintained at 50°C for 3 h with continuous stirring. At the end of the reaction, the mixture was cooled, neutralized with NaOH, and treated by adding ethanol. The precipitate was dissolved in DW, dialyzed exhaustively against DW, and then lyophilized (Figure 9) [117]. The study of Lu et al. [117] showed that COS-S with different degrees of substitution protected MIN6 from H_2_O_2_ induced apoptosis; their study suggested that COS-S prevented apoptosis probably by downregulating H_2_O_2_ induced Bax mRNA expression, Caspase-3 mRNA expression, and NF-*κ*B/p65 activation and upregulating Bcl-2 mRNA expression.

Lu et al. [118] evaluated the protective effects of COS-S against hydrogen peroxide-induced damage in MIN6 cells. They tested the effect of COS-S on cell viability, morphology, insulin contents, malondialdehyde (MDA) inhibition, lactate dehydrogenase (LDH) release, and levels of antioxidant enzymes including catalase (CAT), SOD, and glutathione peroxidase (GPx) under oxidative damage by H_2_O_2_. The study of Lu et al. [118] demonstrated that COS-S exhibited antioxidant effect and enhanced the cell viability; it attenuated the production of ROS, MDA, and LDH. Lu et al. [118] suggested that the protective effects of COS-S can be partly attributed to increase of antioxidant enzyme activities and reduction of intracellular ROS, along with the capacity of suppressing MIN6 cell apoptosis.

The antioxidant effect of COS-S on H_2_O_2_ induced oxidative damage in Chang cells was reported by Lee et al. [119]. COS-S was prepared by deacetylating chitin using sodium hydroxide to get chitosan; the chitosan was then hydrolyzed to COS by using lactic acid and UF membrane reactor system. The chitosan and COS were lyophilized and dispersed in DW and anhydrous sodium carbonate, trimethylamine sulfur trioxide was added to it, and the mixture was heated at 65°C until a viscous solution or gel was formed. The mixture was cooled and dialyzed exhaustively against DW and freeze-dried to get sulfated chitosan and COS-S. Lee et al. [119] tested these synthesized sulfated chitosan and COS-S for their protective effects against H_2_O_2_ induced oxidative damage in Chang cells and concluded that sulfated chitosan and COS-S possess potent protective effect on liver and DNA against oxidative stress.

The inhibitory effects of COS-S with different molecular weights on angiotensin I-converting enzyme were reported by Qian et al. [120]. Their study concluded that molecular weights of COS and COS derivatives are important factors of ACE inhibition and medium molecular weight COS-S and low molecular weight COS-S inhibited ACE by specifically binding to the active site of ACE and by competing with its natural substrate.

The effect of COS-S as inhibitor of prolyl endopeptidase (PEP) was reported by Je et al. [121]. PEP is a proline-specific endopeptidase with a serine-type mechanism and hydrolyzes peptide bonds at the carboxyl terminus of prolyl residues; it has been reported to be involved in neurodegenerative disorders. Je et al. [121] synthesized COS with high, medium, and low molecular weight and with different degrees of deacetylation (90, 75 and 50%) which were then converted into their corresponding sulphates. Je et al. [121] reported that medium molecular weight, 50% deacetylated COS-S showed the maximum inhibitory action on PEP.

The anti-HIV-1 activity of low molecular weight COS-S was reported by Artan et al. [122]. They reported that COS-S with molecular weight range between 3 and 5 kDa was most effective in inhibiting HIV-1 replication. At nontoxic concentrations, COS-S exhibited remarkable inhibitory activities on HIV-1 induced syncytia formation, lytic effect, and p24 antigen production. In contrast, unsulfated chitooligosaccharides showed no activity against HIV-1. Furthermore, Artan et al. [122] concluded that COS-S blocked viral entry and virus-cell fusion probably by disrupting the binding of HIV-1 gp120 to CD4 cell surface receptor.

Karadeniz et al. [123] reported the antiadipogenic effect of COS-S in 3T3-L1 adipocytes. Their study suggested that COS-S inhibited the mRNA expressions and protein levels of key adipogenic markers such as peroxisome proliferator activated receptor (PPAR)-*γ* and CCAAT enhancer binding protein (C/EBP)-*α*, thereby highlighting its efficacy in the management of obesity.

Ryu et al. [124] reported the effects of COS-S with different molecular weights on the degradation of articular cartilage through unregulated collagenase expression. Their study suggested that COS-S with molecular weight between 3 and 5 kDa effectively inhibited the expressions of collagenases 1 and 3 and thereby prevented TNF-*α* induced degradation of collagen in human chondrosarcoma cells (SW-1353). They concluded that COS-S prevented collagen degradation by inhibiting collagenases 1 and 3 via suppressing TNF-*α* induced NF-*κ*B signaling.

### 5.5. Phenolic Acid Conjugated COS

Phenolic acids, especially hydroxycinnamic acids such as p-coumaric, caffeic, ferulic, and sinapinic acid, and benzoic acids derivatives such as p-hydroxybenzoic, protocatechuic, vanillic, and syringic acids are natural plant hydrophilic antioxidants [125].

Eom et al. [126] synthesized various derivatives of COS conjugating them with protocatechuic, 4-hydroxybenzoic, vanillic, syringic, p-coumaric, caffeic, ferulic, and sinapinic acids and tested their antioxidant activity. Their studies showed that among all the PA-c-COSs synthesized, protocatechuic acid conjugated COSs, and caffeic acid conjugated COS showed the strongest antioxidant activities.
